# ^11^C-Labeled Pictilisib (GDC-0941) as a Molecular Tracer Targeting Phosphatidylinositol 3-Kinase (PI3K) for Breast Cancer Imaging

**DOI:** 10.1155/2019/1760184

**Published:** 2019-11-03

**Authors:** Na Han, Yaqun Jiang, Yongkang Gai, Qingyao Liu, Lujie Yuan, Yichun Wang, Mengting Li, Yongxue Zhang, Xiaoli Lan

**Affiliations:** ^1^Department of Nuclear Medicine, Union Hospital, Tongji Medical College, Huazhong University of Science and Technology, Wuhan 430022, China; ^2^Hubei Province Key Laboratory of Molecular Imaging, Union Hospital, Tongji Medical College, Huazhong University of Science and Technology, 1277 JieFang Avenue, Wuhan 430022, China; ^3^Department of Nuclear Medicine, The Affiliated Hospital of Qingdao University, Qingdao University, Qingdao 266000, China

## Abstract

Pictilisib (GDC-0941) is an inhibitor of phosphatidylinositol 3-kinase (PI3K), part of a signaling cascade involved in breast cancer development. The purpose of this study was to evaluate the pharmacokinetics of pictilisib noninvasively by radiolabeling it with ^11^C and to assess the usability of the resulting [^11^C]-pictilisib as a positron-emission tomography (PET) tracer to screen for pictilisib-sensitive tumors. In this study, pictilisib was radiolabeled with [^11^C]-methyl iodide to obtain ^11^C-methylated pictilisib ([^11^C]-pictilisib) using an automated synthesis module with a high radiolabeling yield. Considerably higher uptake ratios were observed in MCF-7 (PIK3CA mutation, pictilisib-sensitive) cells than those in MDA-MB-231 (PIK3CA wild-type, pictilisib-insensitive) cells at all evaluated time points, indicating good *in vitro* binding of [^11^C]-pictilisib. Dynamic micro-PET scans in mice and biodistribution results showed that [^11^C]-pictilisib was mainly excreted via the hepatobiliary tract into the intestines. MCF-7 xenografts could be clearly visualized on the static micro-PET scans, while MDA-MB-231 tumors could not. Biodistribution results of two xenograft models showed significantly higher uptake and tumor-to-muscle ratios in the MCF-7 xenografts than those in MDA-MB-231 xenografts, exhibiting high *in vivo* targeting specificity. In conclusion, [^11^C]-pictilisib was first successfully prepared, and it exhibited good potential to identify pictilisib-sensitive tumors noninvasively, which may have a great impact in the treatment of cancers with an overactive PI3K/Akt/mTOR signal pathway. However, the high activity in hepatobiliary system and intestines needs to be addressed.

## 1. Introduction

**Figure 1:**
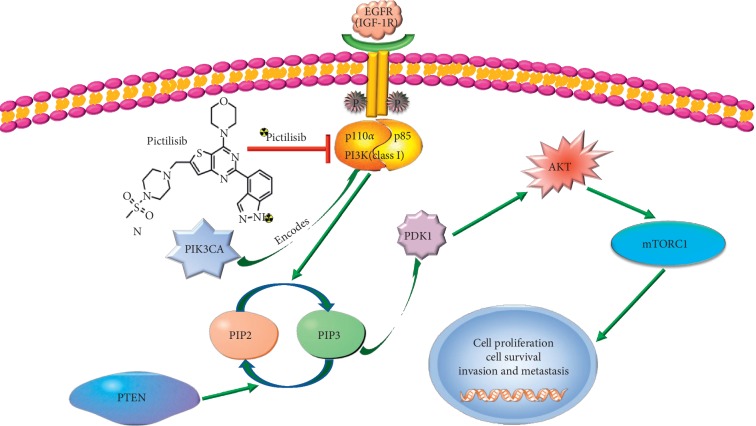
Overview of PI3K/AKT/mTOR signaling pathway and downstream effects. Pictilisib blocks the catalytic activity of PI3K class I isoforms. When pictilisib is labeled with radionuclides, this probe can target and monitor PI3K. Note: the red arrow indicates inhibition and the green arrow indicates promotion. EGFR: epithelial growth factor receptor; IGF-1R: insulin-like growth factor-1 receptor; PIP2: phosphatidylinositol-4,5-bisphosphate; PIP3: phosphatidylinositol-3,4,5-triphosphate; AKT: protein kinase B; mTOR: mammalian target of rapamycin; PTEN: tensin homologue deleted on chromosome 10; PIK3CA: phosphatidylinositol-4,5-bisphosphate 3-kinase catalytic subunit alpha.

The phosphatidylinositol 3-kinase (PI3K) pathway that regulates cell proliferation, survival, and migration is one of the most commonly activated signaling pathways in many human cancers [1]. It can be activated by many cell membrane proteins such as epidermal growth factor receptor (EGFR) and insulin-like growth factor 1 receptor (IGF-1R) [2, 3]. PI3K activation initiates a signal transduction cascade, of which the major effectors are the kinases AKT and mTOR [4] (Figure 1). In a retrospective study, aberrations in the PI3K/AKT/mTOR pathway were identified in 38% of 19784 patients with solid tumors through molecular profiling [5]. Approximately 70% of breast cancers have abnormal activation of PI3K/Akt [6]. The mutation in the PIK3*CA* gene, which encodes the p110*α* catalytic subunit of PI3K, and loss of expression of tensin homology deleted on chromosome ten (PTEN), plays an important role in breast cancer [7, 8]. Previous research reported that PIK3*CA* mutations (exon 9 and/or exon 20) were detected in 45% of primary breast cancers [9]. Meanwhile, some preclinical studies suggested that PIK3*CA* mutations are predictive of sensitivity to PI3K inhibitors [10] and the level of PI3K expression is considered one of the most important prognostic factors for diagnosis and response in solid tumors. In addition, it is reported that aberrant activation of this signaling network may contribute to therapeutic resistance [11]. For example, drug resistance and poor prognosis were associated with abnormal activation of the PI3K pathway among patients with breast cancer treated with trastuzumab [12]. For this reason, the pathway has been an attractive target for cancer therapeutics in recent years, and multiple pharmaceutical companies and academic laboratories are actively developing PI3K inhibitors.

Pictilisib [2-(1H-indazol-4-yl)-6-(4-methanesulfonyl-piperazin-1-ylmethyl)-4-morpholin-4-yl-thieno(3,2-d) pyrimidine] (GDC-0941) (Genentech, Inc., South San Francisco, CA, USA) is an orally bioavailable selective PI3K inhibitor. It has low IC_50_ value of 3 nM against PI3Kp110*α* as well as PI3Kp110*δ* (cell-free assay) [13]. It has been proven to be active in preclinical cells and tumor-bearing mice and is under Phase II clinical trial in patients with advanced solid tumors or lymphoma [14, 15]. It was reported that pictilisib was shown to have good safety and tolerability in Japanese patients with advanced solid tumors [16]. Baird et al. reported that pictilisib was safely administered with a dose-proportional pharmacokinetic profile [17]. Researchers also reported that adding pictilisib to anastrozole significantly increased suppression of tumor cell proliferation in luminal B primary breast cancer [18]. Like most small-molecule inhibitors targeting PI3K, pictilisib binds to the ATP pocket and thus competes with ATP to inhibit the activity of this signaling pathway [13].

In the clinical setting, evaluation of PI3K expression levels requires immunohistochemical detection using patients' tumor samples, which are mainly obtained by biopsy. This is an invasive method and is not suitable for repeating. Molecular imaging analyzes cell function and metabolism at the molecular level and provides opportunities for disease diagnosis, drug efficacy determination, and new drug development [19]. Positron-emission tomography (PET) is one of the most sensitive molecular imaging tools, which is very useful for the noninvasive quantification of *in vivo* biological processes. Previous studies have reported some specific molecular probes for PET imaging of PI3K expression. For example, it was reported that GSK2126458, a PI3K inhibitor, was radiolabeled with both ^11^C and ^18^F [20], but no data regarding their *in vitro* or *in vivo* performance were reported. Another group radiolabeled ZSTK474, another clinical candidate targeting PI3K, with ^18^F to evaluate the PI3K expression levels *in vitro* and *in vivo*. However, the *in vivo* pharmacokinetics of [^18^F]-ZSTK474 were undesirable [21]. It is essential to develop a PI3K/Akt-targeting tracer with better *in vivo* performance for the investigation of this signaling pathway and identification of candidates for pictilisib treatment.

In this study, pictilisib was radiolabeled with ^11^C and applied as a PET tracer to evaluate its pharmacokinetics *in vivo* and to identify pictilisib-sensitive tumors. The aim of this study was to develop a PI3K-targeting tracer for clinical settings to identify candidates for this drug. Herein, we report (1) the radiosynthesis of [^11^C]-pictilisib, (2) the *in vitro* cell assay by various breast tumors, and (3) the *in vivo* micro-PET imaging and biodistribution in breast cancer-xenografted mice. Figure 1 shows the PI3K signaling pathway, the structure of [^11^C]-pictilisib, and the location of [^11^C]-pictilisib in this signaling pathway.

## 2. Materials and Methods

### 2.1. Preparation of [^11^C]-Pictilisib

**Figure 2:**
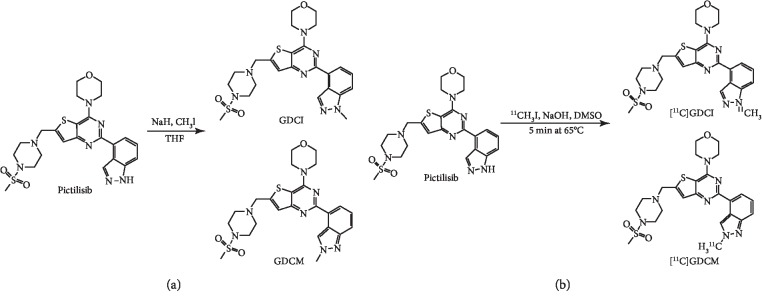
(a) Synthesis of reference standard methyl-pictilisib. (b) Diagram of [^11^C]-pictilisib one-step radiosynthesis.

The precursor pictilisib (Figure 2) was purchased from Selleck Chemicals (Houston, TX, USA). Acetonitrile, ethyl alcohol, and dimethyl sulfoxide were purchased from Sigma-Aldrich (St. Louis, MO, USA) or Fisher Scientific (Pittsburgh, PA, USA). The water used refers to ultrapure water. The filters used to sterilize the PET tracer solution were Millex-GP 0.22 *µ*m (Millipore, Inc., Bedford, MA, USA). A reference standard [^12^C]-pictilisib was synthesized. Radiolabeling of [^11^C]-pictilisib was performed with a radiosynthesis module (GE TraceLab FXC-Pro®; GE Healthcare, Brussels, Belgium). The reaction was stirred and heated to 65°C for 5 min and then cooled to room temperature quickly using liquid nitrogen. The reaction mixture was purified by semipreparative high-performance liquid chromatography (HPLC) (column: Phenomenex Luna 5 *µ*m C18 (2) 100A; 250 × 10 mm; mobile phase: EtOH/H_2_O: 43/57; flow rate: 2.8 ml/min; *λ* = 254 nm). In short, the mixture was diluted with 600 *μ*L of 43% ethanol and then injected into the HPLC loop. The product was passed through a sterile 0.22 *µ*m filter and collected into a sterile vial. Subsequently, the product was analyzed through analytical HPLC (column: Phenomenex Luna 5 *µ*m C18(2) 100A; 250 × 10 mm; mobile phase: EtOH/H_2_O: 43/57; flow rate: 2.8 ml/min) by coinjecting with standard methyl-pictilisib into the analytical HPLC.

### 2.2. Cell Culture

We followed the methods of Li et al. [22]. The human breast cancer cell lines MCF-7 (PIK3*CA* mutation, pictilisib-sensitive) and MDA-MB-231 (PIK3*CA* wild-type, pictilisib-resistant) were purchased from the Type Culture Collection of the Chinese Academy of Sciences, Shanghai, China. The MCF-7 and MDA-MB-231 cells were cultured in Dulbecco's modified Eagle's medium (DMEM; Gibco, Carlsbad, CA, USA) containing 10% (v/v) fetal bovine serum (FBS; Gibco), 100 U/mL penicillin, and 100 *µ*g/mL streptomycin (Beyotime, Shanghai, China) and cultivated at 37°C in an environment with 5% CO_2._

### 2.3. Western Blot Analysis

MCF-7 and MDA-MB-231 cells were harvested at ∼80% confluence. Cells were lysed in lysis buffer (Servicebio, Wuhan, China), and the protein concentration was determined using a bicinchoninic acid (BCA) protein assay kit (Servicebio, Wuhan, China). After denaturation, equal amounts of protein (40 *µ*g) were resolved by sodium dodecyl sulfate polyacrylamide gel electrophoresis (SDS-PAGE). The proteins were then transferred to a polyvinylidene fluoride (PVDF) membrane. The blots were incubated with specific primary antibody (anti-phospho-Akt (Ser473) rabbit monoclonal antibody, diluted 1 : 1000; Clone#RM251, Boster Biological Technology, Wuhan, China. Catalog #P00024-6), followed by secondary antibody (goat anti-rabbit, diluted 1 : 3000; ABclonal Technology), then using beyo-enhanced chemiluminescence (beyoECL) plus (Beyotime) to detect the bands. The film was analyzed by using AlphaEase FC software (Alpha Innotech Corporation, San Leandro, CA).

### 2.4. Growth Inhibition Assay

MCF-7 and MDA-MB-231 cells were plated in 96-well plates at a density of 5 × 10^3^ per well. After 6–10 h, cells were treated with pictilisib and [^12^C]-pictilisib at the following concentrations: 100 *µ*M, 50 *µ*M, 20 *µ*M, 10 *µ*M, 1 *µ*M, and 0.1 *µ*M. The vehicle wells and blank wells (without cells) were suspended in complete medium. Each concentration was placed into five wells. Each well was incubated with 10 *µ*L of Cell Counting Kit 8 solution (Dojindo Laboratories, Tokyo, Japan) at 37°C for 1 h (MDA-MB-231) or 0.5 h (MCF-7). Cell viability was measured by absorbance of the generated formazan optical density (OD) at a wavelength of 450 nm after 48 h. The cell survival rate was expressed as

(1) $$\begin{array}{c} \frac{\left(\text{OD value of the experimental group}-\text{OD value of the blank group}\right)}{\left(\text{OD value of the control group}-\text{OD value of the blank group}\right)}\times 100\%. \end{array}$$

The one-half maximal inhibitory concentration (IC_50_) was calculated using commercial software (Prism Version 5; GraphPad Software, La Jolla, CA, USA).

### 2.5. *In Vitro* Serum Stability of [^11^C]-Pictilisib

[^11^C]-Pictilisib (11.1 MBq, 300 *µ*Ci) was mixed with 300 *µ*L of human serum, and the mixture was incubated at 37°C. Aliquots were taken at 30, 60, and 90 min. Plasma protein was precipitated with 100 *µ*L acetone and centrifuged (10000 rpm, 5 min). The solution was analyzed using HPLC under the mobile phase of 20% MeCN/water containing 0.1% trifluoroacetic acid.

### 2.6. Octanol/Water Partition Coefficient

[^11^C]-Pictilisib (370 kBq, 10 *µ*Ci) was added to a mixed solution of phosphate-buffered saline (PBS) (pH 7.4, 0.5 mL) and 1-octanol (0.5 mL) in a 5 mL centrifuge tube. The mixed solution was vigorously vortexed for 5 min and centrifuged (3000 rpm, 5 min). 100 *µ*L of samples from each phase was quantified for radioactivity using a *γ*-counter. The partition coefficient was calculated as follows: Log *P* = Log 10 (counts in 1 − octanol/counts in PBS).

### 2.7. Internalization Assay

To determine the binding efficacy of [^11^C]-pictilisib, MCF-7 and MDA-MB-231 cells were used. Cells were seeded into 24-well (three wells of each sample) plates at 37°C and cultured for 24 h. The cells were then incubated with 100 *µ*L (5 *µ*Ci) of [^11^C]-pictilisib for 10, 30, and 60 min at 37°C. Then, the medium was removed and the cells were washed three times with ice-cold PBS. After that, to collect the surface-bound fraction, each well was treated with 800 *µ*L of 20 mM acetate-HBSS (Hanks' Balanced Salt Solution, pH 4.0) and incubated at 37°C for 10 min. After removal of the surface-bound fraction, the cells were washed with PBS. Then, the cells were lysed with 1 N NaOH at 37°C for 5 min. The radioactivity in the medium, surface-bound fraction, and cell lysate was measured with an automatic gamma counter (2470 WIZARD PerkinElmer, Waltham, MA, USA).

### 2.8. Animal Models

Female BALB/c nude mice (4 weeks old, 15–17 g) and female BALB/c mice (6 weeks old, 22–25 g) (Beijing HFK Bioscience Co., Ltd., Beijing, China) were kept in a specific pathogen-free barrier system room with constant ambient temperature under a 12 h/12 h light/dark cycle. All animal experiments were performed according to the guidelines approved by the Institutional Animal Care and Use Committee of Tongji Medical College, Huazhong University of Science and Technology. For xenograft tumor models, BALB/c nude mice were subcutaneously injected in the right upper front leg with either MDA-MB-231 cells (1 × 10^7^ in 150 *µ*L PBS) or MCF-7 cells (5 × 10^6^ in 150 *µ*L PBS). Tumors were grown 14 to 21 days until the maximal dimension reached 0.8∼1.0 cm for PET imaging.

### 2.9. Micro-PET Imaging

Dynamic micro-PET imaging was performed on non-tumor-bearing BALB/c mice using a micro-PET scanner (BioCaliburn LH; Raycan Technology Co., Ltd., Suzhou, China). Mice were anesthetized with 1% pentobarbital sodium by intraperitoneal injection, starting 10 min before injection. [^11^C]-Pictilisib (approximately 3.7 MBq) was injected into mice via the tail vein, and approximately 30 seconds later dynamic scans were acquired (6 × 10 s, 4 × 30 s, 4 × 60 s, 4 × 2 min, 3 × 5 min, 3 × 10 min, a total of 24 frames).

Micro-PET imaging was also performed on BALB/c nude mice bearing MCF-7 and MDA-MB-231 tumors. For static imaging, MCF-7-bearing mice (*n* = 3) and MDA-MB-231-bearing mice (*n* = 3) were injected with [^11^C]-pictilisib (mean dose 7.4 MBq) via the tail vein and images were acquired after 1 h.

Images were reconstructed using a two-dimensional ordered subset expectation-maximization algorithm. A region of interest (ROI) was drawn over the tumor on decay-corrected whole-body coronal images, and lateral muscle selected for the background.

### 2.10. Biodistribution Studies

The MCF-7 (*n* = 3) and MDA-MB-231 (*n* = 3) tumor-bearing mice were each administered 3.7–5.55 MBq of [^11^C]-pictilisib via the tail vein. The mice were sacrificed and dissected at 10, 30, and 60 min after injection. Blood, brain, liver, spleen, kidney, stomach, small intestines, colon, bone, muscle, whole tail, and tumors were all collected, washed, and weighed, particularly care should be taken to clean the residual blood and intestinal lumen contents of tissues. The radioactivity was measured with an automatic *γ*-counter. Radioactivity for each tissue was expressed as a percentage of the injected dose per gram of wet tissue (%ID/g). All radioactivity outcomes were corrected for decay.

### 2.11. Immunohistochemical Staining

The tumor tissues were collected for immunohistochemistry (IHC) study after PET imaging. Tumors were removed, dissected, and immersed in 4% paraformaldehyde in PBS (pH 7.4) for 24 h to prepare frozen sections. For immunohistochemical staining, 5 *µ*m tumor sections were processed with a sliding microtome (Micom, Walldorf, Germany). The slides were rinsed with PBS and blocked with 3% BSA for 30 min followed by incubation with primary antibody (anti-phospho-Akt (Ser473) rabbit monoclonal antibody, diluted 1 : 200; Clone#RM251; Boster Inc., Wuhan, China) overnight at 4°C. The sections were rinsed three times with PBS (pH 7.4) for 5 min and treated in horseradish peroxidase- (HRP-) conjugated secondary antiserum for 50 min at 37°C. Then, they were washed thoroughly and treated in diaminobenzidine (DAB) for 3–5 min until a brown reaction product was observed.

### 2.12. Statistical Analysis

All data are expressed as mean ± standard deviation. The differences between two groups were compared by a two-tailed *t*-test. *P* values <0.05 were considered statistically significant between groups.

## 3. Results

### 3.1. Characteristics of Reference Standard and Growth Inhibition Assay

Two compounds were obtained after pictilisib methylation using methyl iodide. They were isomers and were named as GDCI (4-(2-methyl-1H-indazol-4-yl)-6-((4-(methylsulfonyl) piperazin-1-yl)thieno [3,2-d]pyrimidin-4-yl)morpholine) and GDCM (4-(2-methyl-2H-indazol-4-yl)-6-((4-(methylsulfonyl)piperazin-1-yl)thieno [3,2-d]pyrimidin-4-yl)morpholine) (Figure 2(a)). The characteristics of the reference standards were identified by ^1^H-NMR and ^13^C-NMR ([Supplementary-material supplementary-material-1]). The results of NMR spectroscopy were also listed in the supplement data ([Supplementary-material supplementary-material-1]).

Their molecular weights were 527.66 g/mol. As illustrated in Table 1 and [Supplementary-material supplementary-material-1], GDCM exhibited a significantly higher IC_50_ value compared with GDCI for both MCF-7 cells and MDA-MB-231 cells. Therefore, GDCI was selected for further evaluation as a PI3K-imaging tracer.

### 3.2. Characteristics of the Radiochemical

**Figure 3:**
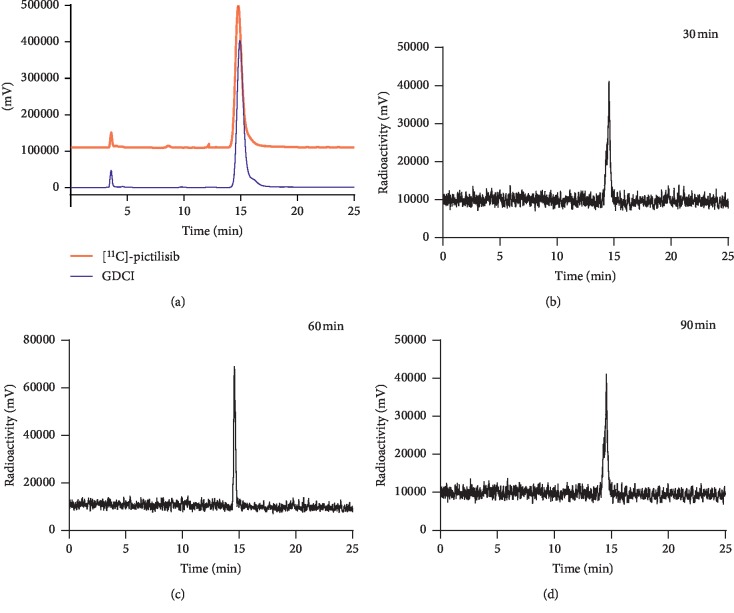
(a) Analytic HPLC chromatograms after the coinjection of [^11^C]-pictilisib (retention time = 14.05 min) and reference standard GDCI (UV 254 nm); (b)–(d) [^11^C]-pictilisib stability in human serum after incubation at 37°C for 30 min, 60 min, and 90 min, respectively.

As shown in Figure 2(b), when pictilisib reacted with [^11^C]-methyl iodide (^11^CH_3_I), two compounds were produced, namely, [^11^C]-GDCI and [^11^C]-GDCM. The products were identified and separated to be [^11^C]-GDCI and [^11^C]-GDCM by semipreparative HPLC ([Supplementary-material supplementary-material-1]). As we have mentioned before, GDCI was selected as the PI3K-imaging probe because of its low IC_50_ value. We considered [^11^C]-GDCI as our product and defined it as “[^11^C]-pictilisib” for purposes of discussion, and it was confirmed by analytic HPLC (Figure 3).

The whole labeling process was completed within 40–45 min from the end of bombardment, including purification with HPLC. The decay-corrected radioactivity yield was 33.7 ± 9.3% (*n* = 6) based on the total [^11^C]-CO_2._ The radiochemical purity of [^11^C]-GDCI was >95%, as measured by analytic HPLC. The specific activity was 52.2 ± 17.0 GBq/*µ*moL (*n* = 6). [^11^C]-Pictilisib was stable in plasma with a radiochemical purity of >95% for 90 min (Figures 3(b)–3(d)). The Log *P* value of [^11^C]-pictilisib was 1.96 ± 0.05 (*n* = 5).

### 3.3. Cell and Xenograft Characterization

**Figure 4:**
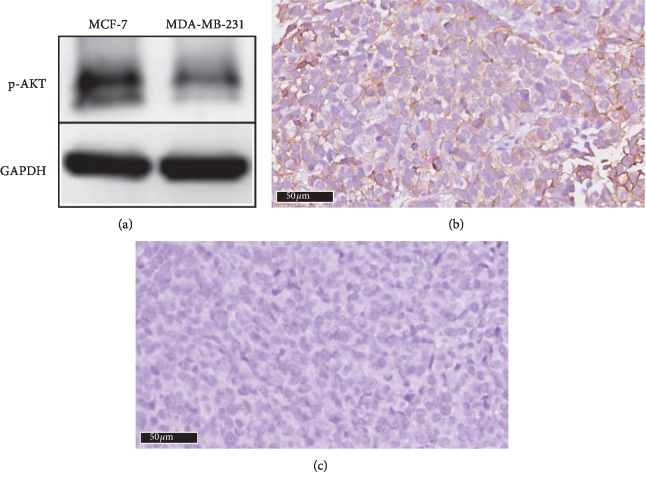
Western blotting (a) for the two breast cancer cells and immunohistochemical staining for tumor tissues. (b) MCF-7 tumor and (c) MDA-MB-231 tumor (×400).

As we mentioned before, the downstream PI3K signaling pathway phosphorylation of AKT will be affected if PI3K was activated. The western blotting results indicated that MCF-7 cells expressed high levels of pAKT than MDA-MB-231 cells (Figure 4(a)). For the IHC staining, MCF-7 xenografts showed the same tendency, which was consistent with the western blotting results (Figures 4(b) and 4(c)).

### 3.4. *In Vitro* Cell Study

**Figure 5:**
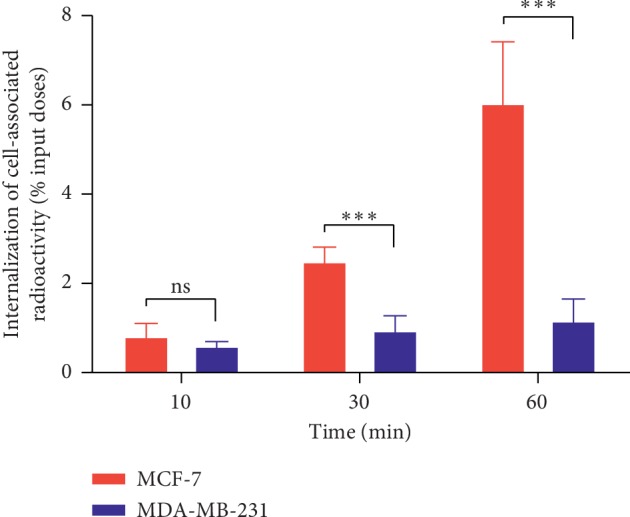
*In vitro* cell study. Cell internalization of [^11^C]-pictilisib in MCF-7 and MDA-MB-231 breast cancer cells at 10 min, 30 min, and 60 min. The internalization rate between MCF-7 and MDA-MB-231 cells was statistically significantly different at 30 min and 60 min (*n* = 3, mean ± SD) (^*∗∗∗*^*P* < 0.001, ns means *P* > 0.05).

Figure 5 shows the internalization of [^11^C]-pictilisib in MCF-7 and MDA-MB-231 cells at 10, 30, and 60 min. The internalization rate of the two cell types was 0.83 ± 0.27 vs. 0.62 ± 0.08% (*P*=0.175), 2.51 ± 0.30 vs. 0.97 ± 0.31% (*P* < 0.001), and 6.06 ± 1.36 vs. 1.19 ± 0.47% (*P* < 0.001) at 10, 30, and 60 min for MCF-7 and MDA-MB-231 cells, respectively.

### 3.5. Micro-PET Imaging with [^11^C]-Pictilisib in Normal Mice and Breast Cancer-Xenografted Mice

**Figure 6:**
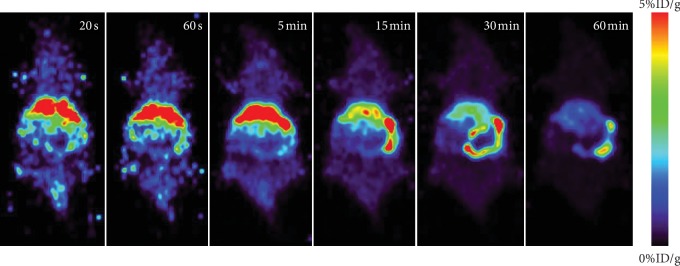
Dynamic micro-PET imaging in normal BALB/c mouse from 0 to 60 min (dose approximately 3.7 MBq). Hepatobiliary excretion and intestinal reuptake were the main metabolic pattern for [^11^C]-pictilisib *in vivo*.

Dynamic microimaging was performed to evaluate the *in vivo* uptake of [^11^C]-pictilisib in normal BALB/c mice from 0 to 60 min after tracer injection. The liver and small intestine showed high uptake of [^11^C]-pictilisib. [^11^C]-Pictilisib is mainly accumulated in the liver in the early phase, then gradually discharged into the duodenum through the common bile duct through the bile over time, and finally the small intestine showed high uptake of the probe in the later phase. Little activity was found in the brain (Figure 6).

**Figure 7:**
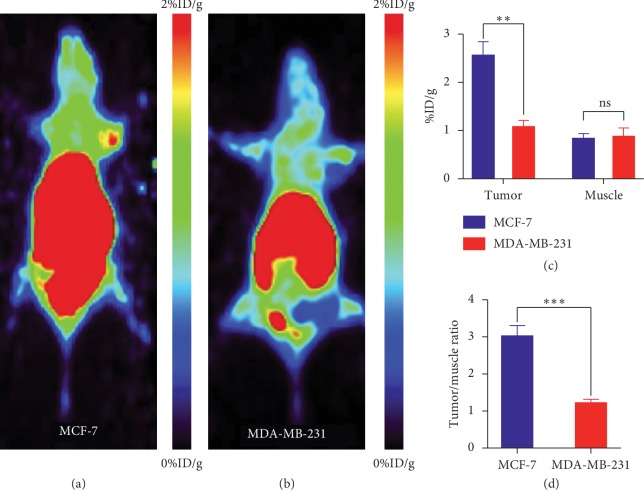
Static micro-PET imaging in MCF-7-bearing mouse and MDA-MB-231-bearing mouse 60 min after injection. (a) The MCF-7 xenograft displayed evident uptake of [^11^C]-pictilisib. (b) No significant accumulation in the MDA-MB-231 xenograft (white arrow). (c) Comparison of tumor and muscle uptakes of [^11^C]-pictilisib in MCF-7 and MDA-MB-231 tumor-bearing mice (^*∗∗*^*P* < 0.01). (d) Comparison of tumor-to-muscle ratio in MCF-7 and MDA-MB-231 tumor-bearing mice (^*∗∗∗*^*P* < 0.001).

Static micro-PET imaging was performed to assess the tumor-targeting potential of [^11^C]-pictilisib. The scan was performed 60 min after injection. MCF-7 tumors displayed evident uptake of [^11^C]-pictilisib, while no obvious accumulation was observed in the MDA-MB-231 xenografts throughout the measurement period of 60 min. As shown in Figure 7, the ROI values of MCF-7 and MDA-MB-231 tumors were 2.58 ± 0.23%ID/g and 1.10 ± 0.10%ID/g, while the lateral muscle were 0.85 ± 0.07%ID/g and 0.90 ± 0.13%ID/g, and the value of T/M was 3.04 ± 0.22 and 1.23 ± 0.08, respectively. Much difference could be seen between the uptakes of MCF-7 and MDA-MB-231 tumors and T/M ratios (*P* < 0.01 and *P* < 0.001, respectively). However, relatively high background was observed throughout the body in MCF-7 tumor-bearing mice. In addition, the liver and intestines showed high uptake of [^11^C]-pictilisib in xenografted and normal mice (Figure 7).

### 3.6. Biodistribution Studies

**Figure 8:**
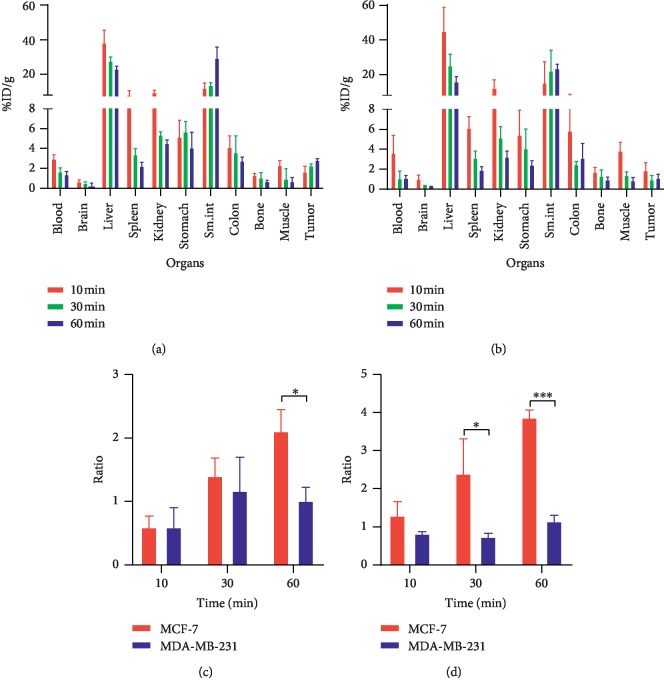
Biodistribution of [^11^C]-pictilisib in tumor xenografts. (a) and (b) Uptake of [^11^C]-pictilisib in MCF-7 and MDA-MB-231 tumors was measured at 10, 30, and 60 min after injection. (c) and (d) Comparison of tumor/blood and tumor/muscle ratios between MCF-7 and MDA-MB-231 tumors at 10, 30, and 60 min after injection. Data are expressed as mean ± SD (*n* = 3). (^*∗*^*P* < 0.05, ^*∗∗∗*^*P* < 0.001).

As shown in Figure 8, the liver took up the most radioactivity in the early phase (10 min) with 38.49 ± 5.71%ID/g vs. 45.28 ± 10.99%ID/g in all mice, while small intestine increased over time and contained the most activity in the later phase (60 min) with 29.59 ± 4.98%ID/g vs. 23.81 ± 1.77%ID/g, which indicated the hepatobiliary excretion and intestinal reuptake of [^11^C]-pictilisib. Small amounts of radioactivity (<1.5%ID/g) were distributed in the brain, which was consistent with the results of dynamic imaging, suggesting that the drug did not pass the blood-brain barrier to a large extent.

In MCF-7-bearing mice, uptake in the tumor increased over time and peaked at 60 min (2.88 ± 0.07)%ID/g. [^11^C]-Pictilisib showed rapid distribution in the blood pool (2.99 ± 0.32 %ID/g) at 10 min after injection, with rapid clearance with 1.40 ± 0.22%ID/g remaining at 60 min. The accumulation of [^11^C]-pictilisib in MCF-7 xenografts was significantly higher than that in MDA-MB-231 xenografts at 60 min (*P* < 0.001). In addition, the ratios of tumor/blood and tumor/muscle in MDA-MB-231-bearing mice at 60 min were also significantly lower than those of MCF-7 (*P* < 0.05 and *P* < 0.001, respectively).  Table 2 shows the biodistribution of [^11^C]-pictilisib in organs and tumors.

## 4. Discussion

Molecular targeted therapies have many advantages in cancer treatment, of which small molecule targeted drugs are widely studied in preclinical and clinical trials [23]. Pictilisib, a PI3K inhibitor, is a small molecule which is under clinical trial. In this study, we successfully synthesized [^11^C]-pictilisib, a PET radiotracer, that targets PI3K for screening pictilisib-sensitive tumors. To the best of our knowledge, this is the first investigation for the feasibility of radiolabeling pictilisib with ^11^C as a molecular probe for breast cancer. The pharmacokinetics of [^11^C]-pictilisib and its targeting ability for breast cancer were evaluated *in vitro* and *in vivo* in this study, with the aim of providing an approach to select the appropriate candidates for pictilisib therapy.

Currently, small-molecule inhibitors are widely used in the clinic and more are being studied in clinical trials. Providing evidence for an inhibitor's effectiveness in patient subgroups for clinical trials and eventual therapy is very important to choose the right patients in advance. We have synthesized a probe to estimate whether the drug can accumulate in tumor by [^11^C]-pictilisib PET imaging, thus expecting to achieve that purpose.

We demonstrated the pharmacokinetics of pictilisib. A rapid and predominant hepatobiliary elimination of [^11^C]-pictilisib was observed in animal models on micro-PET images. In normal BALB/c mice, the liver showed the highest uptake of the radiotracer in early imaging (10 min). Subsequently, the activity in small intestine increased over time and it became the organ with the highest uptake by 60 min. The mode of clearance hindered the evaluation of areas around the abdomen and pelvis, which may make it difficult to show tumor foci in these sites. Some previous studies had also observed a similar pattern for other radiolabeled protein inhibitors tested in mice [24–26].

We further demonstrated that [^11^C]-pictilisib was efficient to distinguish pictilisib-sensitive tumors as a molecular probe via *in vitro* cell uptake studies and *in vivo* micro-PET scans on MCF-7- and MDA-MB-231-bearing mice. For *in vitro* cell uptake studies, we assessed the uptake of [^11^C]-pictilisib by breast cancer cells, since pictilisib binds to an intracellular target and reaches this target by passive diffusion through the cell membrane. Pictilisib-sensitive MCF-7 showed a significantly higher uptake ratio over time than pictilisib-insensitive MDA-MB-231. In micro-PET scanning of tumor-bearing mice, the xenografts of MCF-7 could be clearly visualized, although high radioactivity was distributed throughout the abdomen, whereas xenografts of MDA-MB-231 cells were not visible. For biodistribution experiments, the clearance pattern was in keeping with the images of micro-PET scanning.

Labeling with ^11^C does not change the elemental composition of pictilisib, and the labeling method of ^11^C is relatively simple. However, there are some findings that are unfavorable for imaging. First, as a fat-soluble small molecule, the hepatobiliary excretion and intestinal reuptake patterns of pictilisib result in very slow metabolism of probe from the liver and small intestine, which does not match the short half-life of ^11^C. Second, as pictilisib had to cross the cell membrane and to compete with high intracellular ATP concentration for receptor binding, it may result in the insufficient intratumoral concentrations of [^11^C]-pictilisib. Last but not the least, the tumor uptake achieved better image at 1 h after injection of [^11^C]-pictilisib, which is not suitable for the short half-life of ^11^C. Other researchers have encountered the same problems when ^11^C labeling small molecules. Ortu et al. [27] synthesized [^11^C]-ML03 and evaluated the probe *in vitro* and *in vivo*. They observed moderate uptake in tumor and high uptake in the liver and intestine. However, they found that the trend of the tumor/blood ratios suggested that this ratio might improve over longer times. Zhang et al. [24] labeled gefitinib with ^11^C and found that its high lipophilicity (log P 3.6) resulted in distribution throughout the body and displayed a slow decrease of uptake from the liver and small intestine. Memon et al. [26] labeled erlotinib with ^11^C and also found that activity was mainly accumulated in the liver and small intestine 1 h after [^11^C]-erlotinib injection.

To reduce the high background and to improve the potency of [^11^C]-pictilisib for imaging, the following aspects could be considered. First, its high lipophilicity may be behind the high accumulation in the hepatobiliary system and its high excretion rate into the small intestine. Hence, to optimize the pharmacokinetics, improving water solubility may be required. Xu et al. [28] modified the 3-position of pyridine with a short chain polyethylene glycol and labeled it with ^18^F to produce ^18^F-PEG_3_-FPN, which had higher labeling efficacy and better *in vivo* pharmacokinetics along with lower liver uptake compared with ^18^F-5-FPN [29]. Second, the half-life of ^11^C is short (approximately 20 min), so a radionuclide with a longer half-life, such as ^18^F (approximately 110 min) or ^68^Ga (approximately 67.6 min), may be needed to prolong the imaging time. Another team has developed [^18^F]-afatinib as a new tyrosine kinase inhibitor PET tracer for EGFR-positive tumors. They suggested that the tumor showed good retention with around 1%ID/g remaining at 120 min, which led to moderate/high tumor-to-blood and high tumor-to-muscle ratios [30].

In conclusion, we developed a PI3K-targeting tracer [^11^C]-pictilisib based on a phase II clinical trial and evaluated its pharmacokinetics *in vivo*. Our data demonstrated that [^11^C]-pictilisib has high specificity for PI3K and can be used as a noninvasive method for imaging PI3K expression and the identification of pictilisib-responding tumors. However, the high background in the liver and intestines is problematic. We are exploring solutions to improve this tracer's imaging qualities.

## Figures and Tables

**Table 1:** IC_50_ values of pictilisib, GDCI, and GDCM against cells.

Compounds	MCF-7		MDA-MB-231	
	IC_50_ (*μ*mol/L)	95% CI	IC_50_ (*μ*mol/L)	95% CI
Pictilisib	7.14	5.56–9.17	19.57	14.40–26.60
GDCI	6.91	5.02–9.51	16.00	15.06–17.00
GDCM	73.26	28.64–187.4	130.8	71.57–406.8

**Table 2:** Biodistribution of [^11^C]-pictilisib in BALB/C nude mice bearing MCF-7 and MDA-MB-231 xenografts (%ID/g mean ± SD).

Organ (%ID/g)	MCF-7 (*n* = 3)			MDA-MB-231 (*n* = 3)		
	10 min	30 min	60 min	10 min	30 min	60 min
Blood	2.99 ± 0.32	1.69 ± 0.28	1.40 ± 0.22	3.66 ± 1.40	1.09 ± 0.58	1.15 ± 0.18
Brain	0.70 ± 0.10	0.57 ± 0.04	0.23 ± 0.22	1.06 ± 0.29	0.44 ± 0.03	0.35 ± 0.03
Liver	38.49 ± 5.71	27.98 ± 1.61	23.42 ± 0.93	45.28 ± 10.99	25.37 ± 5.35	16.19 ± 2.33
Spleen	8.20 ± 1.94	3.43 ± 0.46	2.26 ± 0.26	6.15 ± 0.91	3.14 ± 0.54	1.92 ± 0.27
Kidney	9.82 ± 0.82	5.41 ± 0.22	4.56 ± 0.25	12.59 ± 3.73	5.19 ± 0.87	3.25 ± 0.45
Stomach	5.22 ± 1.33	5.71 ± 0.83	4.12 ± 1.25	5.46 ± 2.01	4.11 ± 1.55	2.45 ± 0.32
Sm. int	12.32 ± 2.09	13.93 ± 1.05	29.59 ± 4.98	15.57 ± 9.66	22.37 ± 9.50	23.81 ± 1.77
Colon	4.15 ± 0.91	3.65 ± 1.31	2.79 ± 0.29	5.89 ± 2.3	2.51 ± 0.20	3.14 ± 1.18
Muscle	1.35 ± 0.10	1.10 ± 0.37	0.74 ± 0.04	1.74 ± 0.35	1.35 ± 0.48	1.00 ± 0.15
Bone	2.33 ± 0.35	0.98 ± 0.81	0.73 ± 0.27	3.86 ± 0.66	1.41 ± 0.26	0.89 ± 0.21
Tumor	1.72 ± 0.38	2.31 ± 0.12	2.88 ± 0.07	1.90 ± 0.62	0.98 ± 0.32	1.15 ± 0.26

Uptake ratio						
Tumor-to-blood	0.59 ± 0.18	1.41 ± 0.28	2.11 ± 0.34	0.60 ± 0.31	1.17 ± 0.52	1.01 ± 0.21
Tumor-to-muscle	1.30 ± 0.36	2.41 ± 0.91	3.87 ± 0.20	0.83 ± 0.05	0.74 ± 0.09	1.15 ± 0.15

## Data Availability

The data used to support the findings of this study are available from the corresponding author upon request.

## Supplementary Materials

Supplementary Materials Figure S1: ^1^H-NMR and ^13^C-NMR spectra of GDCI and GDCM. Figure S2: normalized dose response of the drug for the two cell lines. Figure S3: semipreparative radio high-performance liquid chromatography (radio-HPLC) of [^11^C]-pictilisib.
