# Supplementary material for: 11C-Labeled Pictilisib (GDC-0941) as a Molecular Tracer Targeting Phosphatidylinositol 3-Kinase (PI3K) for Breast Cancer Imaging
Source: Contrast Media Mol Imaging. 2019 Nov 3;2019:1760184. doi: 10.1155/2019/1760184 (PMC6877939; doi:10.1155/2019/1760184)
Supplement: Supplementary Materials — Figure S1: 1H-NMR and 13C-NMR spectra of GDCI and GDCM. Figure S2: normalized dose response of the drug for the two cell lines. Figure S3: semipreparative radio high-performance liquid chromatography (radio-HPLC) of [11C]-pictilisib. [file 1760184.f1.pdf]

Title Page

Title: ^{11}C Labeled Pictilisib (GDC-0941) as a Molecular Tracer Targeting Phosphatidylinositol 3-kinase (PI3K) for Breast Cancer Imaging

Authors: Na Han,[1,2,3] Yaqun Jiang,[1,2] Yongkang Gai,[1,2] Qingyao Liu,[1,2] Lujie Yuan,[1,2] Yichun Wang,[1,2] Mengting Li,[1,2] Yongxue Zhang,[1,2] Xiaoli Lan,[1,2]*

Author information

[1]*Department of Nuclear Medicine, Union Hospital, Tongji Medical College, Huazhong University of Science and Technology, Wuhan 430022, China*

[2]*Hubei Key Laboratory of Molecular Imaging, Wuhan 430022, China*

[3]*Department of Nuclear Medicine，The Affiliated Hospital of Qingdao University, No.16 JiangSu Road, Qingdao, Shandong Province 266000, China*

***Corresponding author:** Xiaoli Lan, M.D, Ph.D.

Address: Department of Nuclear Medicine, Union Hospital, No. 1277 Jiefang Ave, Wuhan, Hubei Province 430022, China.

Tel.: +86-27-83692633(O), +86-13886193262(mobile); Fax: +86-27-85726282.

E-mail: LXL730724@hotmail.com

Funding

This work was supported by the National Natural Science Foundation of China (No. 81630049 and 81771863).

Conflicts of interest

The authors declare no potential conflicts of interest.

Supplement data

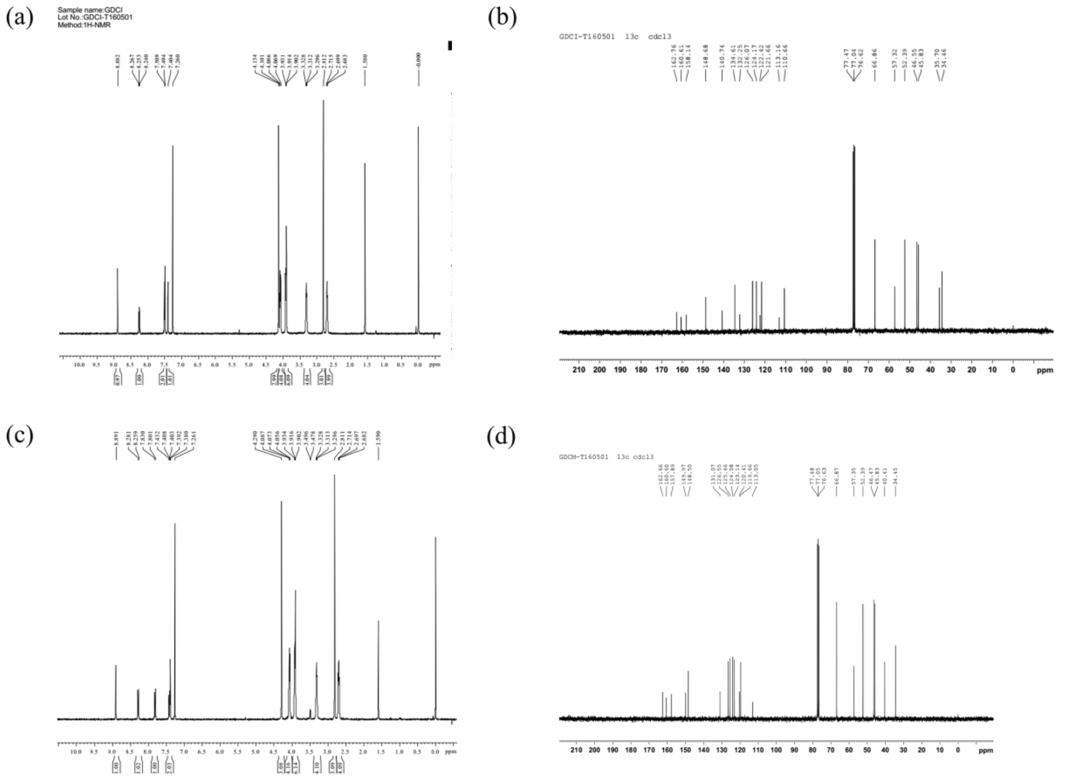

Figure S1: (a) and (b) showed the ^{1}H-NMR and ^{13}C-NMR of GDCI. (c) and (d) showed the ^{1}H-NMR and ^{13}C-NMR of GDCM.

NMR spectroscopy:

GDCI ^{1}HNMR(300MHz,$CDCl_3$) :

δ8.882(s,1H),δ8.253(t,J=4.05Hz,1H),δ7.494(d,J=4.5Hz,2H),δ7.404(s,1H),δ44.134(s,3H),δ4.086(t,J=4.8Hz,4H),δ3.914(t,J=5.1Hz,4H),δ3.902(s,2H),δ3.312(t,J=4.8Hz,4H), δ2.812(s,3H),δ2.699(t, J=4.8Hz,4H);

GDCI ^{13}C NMR($CDCl_3$):

δ162.76,160.61,158.14,148.68,140.74,134.61,132.25,126.07,124.17,122.42,121.66,113.16,110.66,77.47,77.04,76.62,66.86,57.32,52.39,46.55,45.83,35.70,34.46;

GDCM ^{1}HNMR(300MHz,$CDCl_3$):

δ8.891(s,1H),δ8.259(d,J=6.6Hz,1H),δ7.801(d,J=8.7Hz,1H),δ7.408(dd,J=7.2,8.7Hz,1H),δ7.380(s,1H),δ4.290(s,3H),δ4.073(t,J=4.65Hz,4H),δ3.916(t,J=4.8Hz,4H),δ3.902(s,2H),δ3.313(t,J=4.8Hz,4H), δ2.811(s,3H),δ2.697(t, J=4.8Hz,4H);

GDCM ^{13}CNMR($CDCl_3$):

δ162.66,160.60,157.89,149.97,148.50,131.07,126.55,125.66,124.08,123.14,120.41,119.66,113.05,77.48,77.05,76.63,66.87,57.35,52.39,46.47,45.83,40.41,34.45.

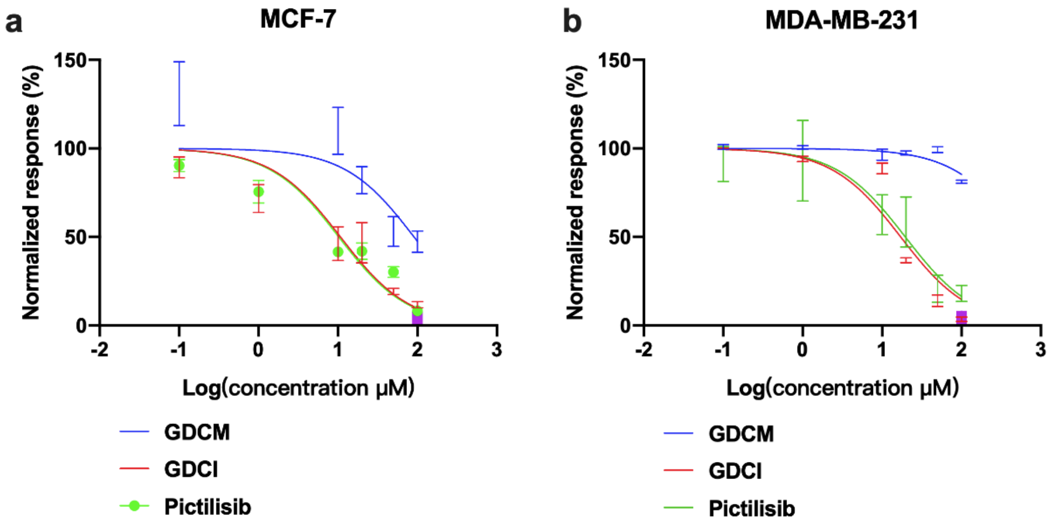

Figure S2 Normalized dose response of the drug for the two cell lines

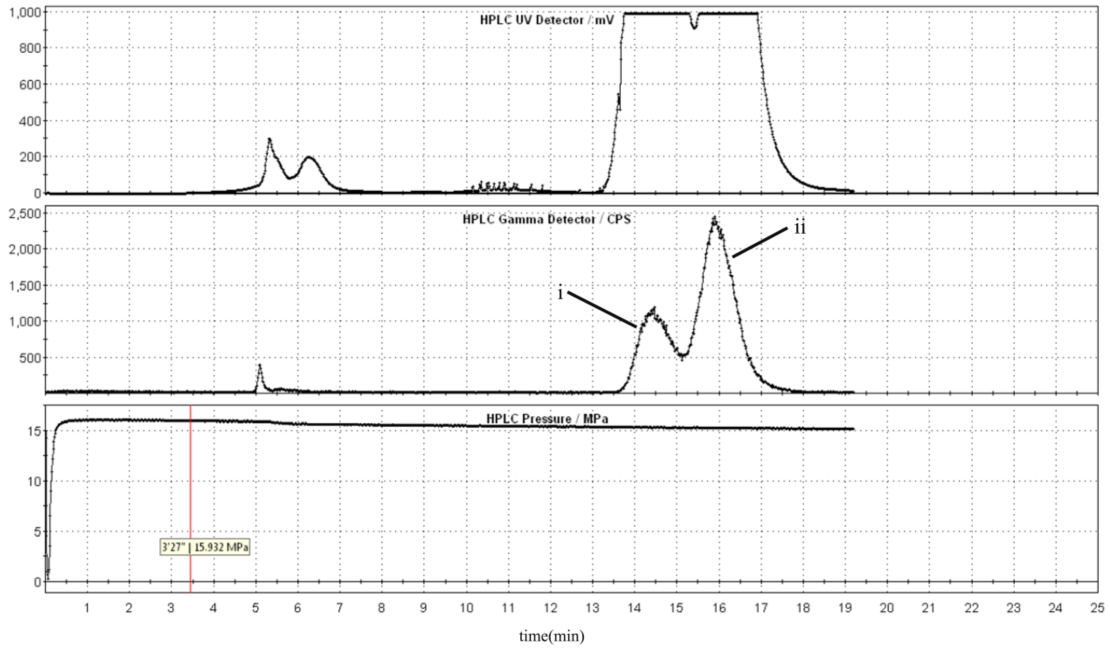

Figure S3: The reaction products were purified by semi-preparative HPLC. ⅰ was [^{11}C]-GDCM and ⅱ was [^{11}C]-GDCI.
